# The role of USP19 in human diseases: from molecular function to clinical relevance

**DOI:** 10.3389/fimmu.2026.1877228

**Published:** 2026-06-26

**Authors:** Qingsong Wang, Haonan Cao, Huan Wei, Xianmin Wang, Tongyong Luo, Jun Yin

**Affiliations:** 1 Department of Pediatrics, West China Hospital Sichuan University Jintang Hospital, Jintang First People’s Hospital, Chengdu, Sichuan, China; 2 Department of Anesthesiology, West China Hospital Sichuan University Jintang Hospital, Jintang First People’s Hospital, Chengdu, Sichuan, China; 3 Department of Public Administration, West China Hospital Sichuan University Jintang Hospital, Jintang First People’s Hospital, Chengdu, Sichuan, China; 4 Pediatric Cardiology Center, Sichuan Provincial Women’s and Children’s Hospital, Chengdu, Sichuan, China; 5 Pediatric Cardiology Center, The Affiliated Women’s and Children’s Hospital of Chengdu Medical College, Chengdu, Sichuan, China; 6 Department of Ultrasound, West China Hospital Sichuan University Jintang Hospital, Jintang First People’s Hospital, Chengdu, Sichuan, China

**Keywords:** biomarker, combination therapy, deubiquitinase, inflammation, signaling pathway, targeted therapy, tumor, USP19

## Abstract

USP19 is an important member of the ubiquitin-specific protease (USP) subfamily within the deubiquitinase superfamily. It primarily regulates protein stability, subcellular localization, and signaling pathway activity by specifically removing ubiquitin modifications from substrate proteins, and it is widely involved in the regulation of cellular physiological homeostasis and various pathological processes. USP19 shows aberrant expression and functional dysregulation in multiple malignancies, participating in the regulation of tumor proliferation, metastasis, apoptosis, immune evasion, and chemoresistance by targeting key molecules such as c-Myc, p53, PD-L1, MGMT, and PARK7. Additionally, it regulates inflammatory responses, immune responses, viral infections, and non-neoplastic diseases such as liver injury, fibrosis, and neurodegeneration. Mechanistic research on USP19 has expanded considerably, and its key substrates and signaling pathways have become potential targets for pharmacological intervention; small-molecule modulators and the development of targeted strategies remain at the preclinical stage. USP19 displays disease-specific expression patterns across different tissues: it is aberrantly overexpressed in most tumors and is closely associated with poor patient prognosis, whereas in certain tumors and non-neoplastic diseases it shows low expression or a protective upregulation. This article systematically summarizes the molecular characteristics, physiological functions, disease-related mechanisms, and clinical translational potential of USP19, to provide a comprehensive overview for its use as a novel diagnostic biomarker, prognostic stratification tool, treatment response predictor, and direct drug target.

## 1 Introduction

USP19 is a modular deubiquitinating enzyme that regulates protein stability, immune signaling, and cellular stress responses through substrate-specific deubiquitination (1). Rossi et al. (2022) previously reviewed the emerging role of USP19 in oncogenesis, with emphasis on breast cancer cell migration and deubiquitinating mechanisms (2). That synthesis did not, however, integrate USP19 functions in non-neoplastic inflammatory diseases, systematically map the functional divergence between ER-anchored and cytosolic isoforms onto disease mechanisms, or critically distinguish preclinical genetic evidence from clinically validated biomarkers. It also did not evaluate the network position of USP19 relative to other deubiquitinases.

This review addresses these gaps by examining USP19 as an immuno-regulatory hub connecting innate immune attenuation with tumor immune evasion, by organizing its pathological functions according to shared molecular mechanisms rather than anatomical sites, and by critically appraising translational claims, distinguishing evidence tiers, and confronting isoform selectivity, functional redundancy, and druggability bottlenecks.

## 2 Biological features of USP19

### 2.1 Molecular characteristics

Ubiquitin-specific protease 19 (USP19) is a member of the ubiquitin-specific protease family within the deubiquitinase superfamily (2). The USP19 gene is located in the human chromosome 3p21.3 region, a locus that frequently undergoes heterozygous deletion in various cancers, suggesting that USP19 may also possess tumor-suppressive functions (3). USP19 is a modular deubiquitinase with a molecular weight of approximately 150 kDa (2). Its N-terminus contains two tandem CHORD-SGT1/P23 domains, termed CS1 and CS2, which not only mediate interactions between USP19 and other proteins but also inhibit the activity of its catalytic core through an intramolecular mechanism (2, 4). Immediately following the CS domains is a highly conserved USP catalytic domain spanning approximately 450 amino acids (5). This catalytic domain adopts a right-hand-like three-dimensional conformation comprising palm, thumb, and finger subdomains; its active center is formed by conserved Cys-box and His-box motifs, and the catalytic triad consists of cysteine (Cys506), histidine (His1157), and an aspartic acid residue, representing the core of USP19’s deubiquitinase activity (2, 6). Embedded within the catalytic domain are a ubiquitin-like domain and a MYND-type zinc finger domain.

**Figure 1:**
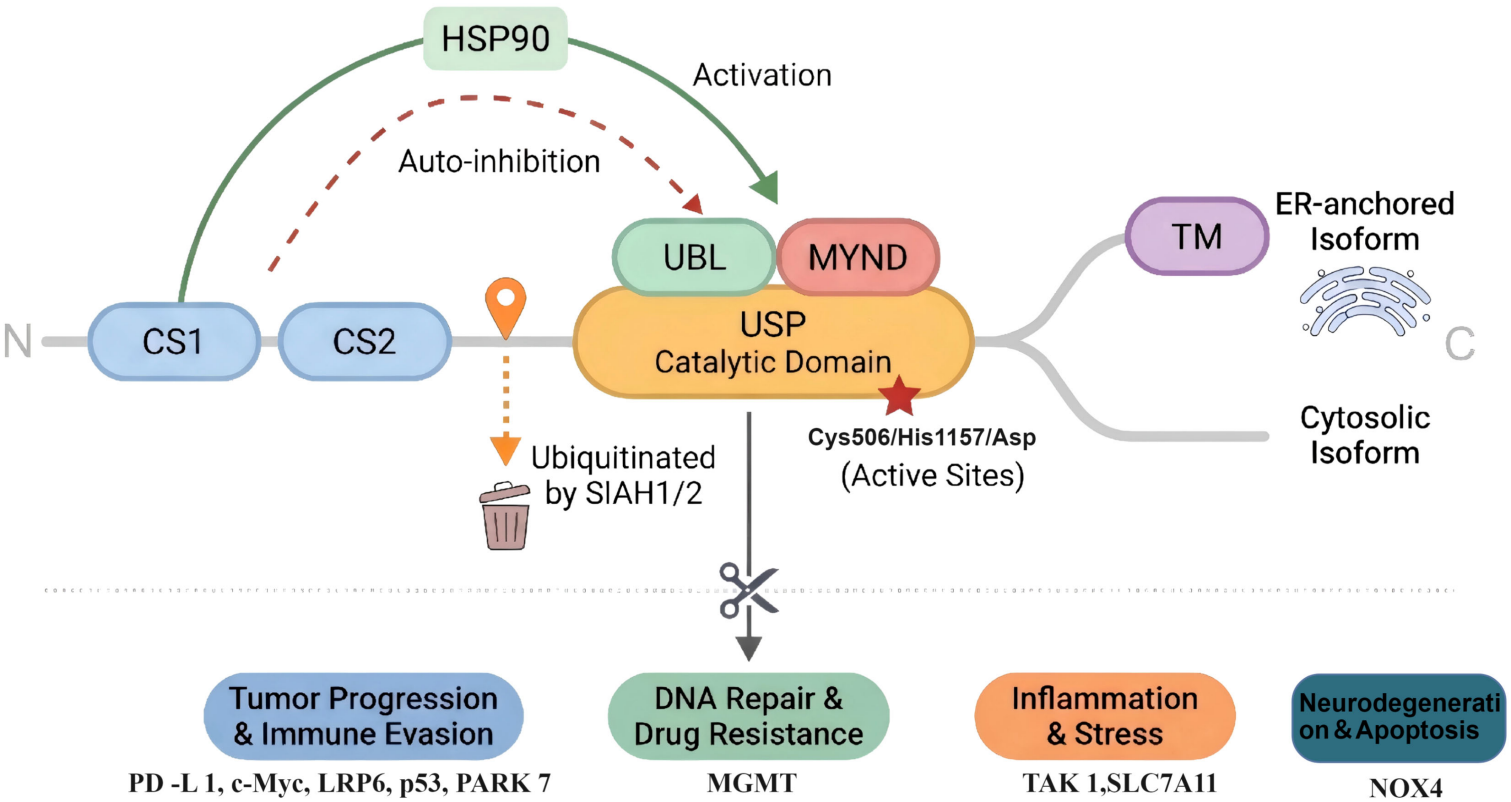
Domain architecture, regulatory mechanisms, and disease-associated substrate spectrum of USP19. The schematic illustrates the N-terminal CS1/CS2 domains, central USP catalytic domain (Cys506/His1157/Asp), UBL/MYND motifs, and the C-terminal transmembrane domain (TM) that distinguishes the ER-anchored isoform from the cytosolic isoform. Upstream regulators (HSP90-mediated activation, SIAH1/2-mediated ubiquitination and degradation, intramolecular autoinhibition) and downstream functional modules (tumor progression and immune evasion, DNA repair and drug resistance, inflammation and stress, neurodegeneration and apoptosis) with representative substrates are annotated.

The USP19 gene produces multiple isoforms through alternative splicing; the most significant structural difference lies at the C-terminus: some isoforms contain a C-terminal transmembrane domain that anchors them to the cytosolic face of the endoplasmic reticulum (ER), making them ER-resident proteins, whereas other isoforms lack this transmembrane domain and remain primarily free in the cytoplasm (2). For example, in clear cell renal cell carcinoma, isoform uc003cvz.3 is the predominant form and is significantly downregulated in advanced tumors (8). Subcellular localization studies have shown that USP19 is distributed in both the nucleus and cytoplasm, with its cytoplasmic localization being largely attributable to ER anchoring (7, 9). Fluorescence co-localization experiments have further confirmed that the cytoplasmic distribution of USP19 exhibits a reticular pattern that overlaps extensively with ER markers (10) (**Figure 1**).

### 2.2 Expression regulatory mechanisms

USP19 expression is precisely regulated at multiple levels to meet the demands of different physiological and pathological states.

At the transcriptional level, the USP19 promoter region contains a binding site for the transcription factor E2F1. Studies in hepatocellular carcinoma have shown that E2F1 can directly bind to the USP19 promoter region, activate its transcription, and thereby upregulate USP19 mRNA and protein levels (11). In skeletal muscle, estrogen receptor α can bind to a functional half-estrogen response element within an intron of the USP19 gene, driving USP19 transcription in response to 17β-estradiol stimulation (12, 13). At the post-translational modification level, the stability and activity of USP19 protein are regulated through multiple mechanisms.

Ubiquitination and degradation regulation: USP19 protein levels are under strict control by the ubiquitin-proteasome system. The E3 ubiquitin ligases SIAH1 and SIAH2 are key negative regulators. SIAH1 directly recognizes and binds the N-terminal amino acid region 462–473 of USP19 through its substrate-binding domain, and catalyzes the formation of K27-linked polyubiquitin chains on lysine residues 489, 490, and 610, thereby targeting USP19 for proteasomal degradation (14, 15). This regulation is of significant importance in antiviral innate immunity: SIAH1 is stabilized in the early phase of viral infection, promoting USP19 degradation to relieve its suppression of immune signaling pathways (14). Similarly, in pathological cardiac hypertrophy models, the transcriptional level of SIAH2 decreases under pressure-overload stimulation, leading to attenuated ubiquitination-mediated degradation of USP19 and consequent accumulation of USP19 protein—a protective feedback mechanism by which cardiomyocytes respond to hypertrophic stimuli (1).

Phosphorylation regulation: USP19 is also a substrate for phosphorylation modifications. Phosphorylation can indirectly regulate USP19 function by influencing substrate recognition. In colorectal cancer, phosphorylation of malic enzyme 1 (ME1) by the ERK2 signaling pathway enhances USP19-mediated deubiquitination and stabilization of ME1, thereby promoting tumor lipogenesis and carcinogenesis (16).

Molecular chaperone regulation: The enzymatic activity of USP19 is regulated by the molecular chaperone HSP90. HSP90 recognizes the N-terminal region of USP19, relieving the intramolecular autoinhibition mediated by the CS domains and thereby activating USP19 deubiquitinase activity (4). In neurodegenerative disease models, this mechanism contributes to USP19-mediated regulation of mutant huntingtin protein aggregation (17, 18).

Subcellular localization regulation: Differences in isoform C-terminal structures leading to ER anchoring versus cytoplasmic release constitute another important dimension of functional regulation. For example, in breast cancer, the ER-anchored USP19 isoform promotes cell migration and invasion, whereas the cytoplasmic isoform lacks this function (19).

### 2.3 Physiological functions

Through its deubiquitinase activity, USP19 removes ubiquitin chains from specific target proteins, thereby reversing protein degradation fates or modulating their signaling functions, and participates in the regulation of a broad spectrum of physiological processes.

Protein homeostasis and cellular stress: As an ER-anchored protein, USP19 is a key participant in the ER-associated degradation (ERAD) pathway and the unfolded protein response (UPR). It can recognize and stabilize specific ERAD substrates, preventing the accumulation and aggregation of misfolded proteins (7, 20). It can also promote the unconventional secretion of misfolded cytosolic proteins (such as α-synuclein and tau protein)—termed the “misfolding-associated protein secretion” pathway—through a catalytic activity-independent mechanism, thereby alleviating stress caused by proteasome dysfunction (21, 22). Furthermore, by stabilizing Beclin-1 and regulating p62 ubiquitination levels, USP19 plays important roles in regulating macroautophagy/autophagic flux, as well as lysosomal protein secretion and lysosomal exocytosis related to the endocytic-lysosomal system (23, 24). USP19 also stabilizes phenylalanine hydroxylase through deubiquitination, regulating phenylalanine metabolism in the liver; loss of this function may lead to metabolic disorders associated with phenylketonuria (9).

Muscle and metabolism: USP19 has been widely established as a key negative regulator of skeletal muscle mass. In various catabolic states—including starvation, diabetes, glucocorticoid treatment, denervation, and tumor cachexia—USP19 expression levels in skeletal muscle are significantly elevated (25, 26). USP19 exerts its pro-atrophic effects by inhibiting myoblast differentiation and promoting degradation of myofibrillar proteins such as myosin (27, 28). USP19 knockout mice demonstrate resistance to denervation- and glucocorticoid-induced muscle atrophy, and can prevent muscle wasting by modulating insulin and glucocorticoid signaling (29, 30). Female-specific regulation of muscle mass is also linked to USP19: 17β-estradiol upregulates USP19 expression through ERα, whereas the soy isoflavone genistein, acting as an ERβ agonist, can antagonize this effect, downregulate USP19, and increase soleus muscle mass (12). Additionally, USP19 participates in adipogenesis; its deficiency impairs lipid accumulation capacity in adipocytes and exacerbates high-fat diet-induced obesity and glucose intolerance (31).

Immune regulation and inflammatory responses: USP19 serves as a pleiotropic negative regulator. Innate immune signaling: USP19 inhibits excessive activation of the type I interferon pathway at multiple nodes. It directly interacts with MAVS, removing its K63-linked ubiquitin chains to suppress MAVS aggregation and downstream signal activation (14). It can also deubiquitinate TRAF3 and TRIF, inhibiting their K63- and K27-linked ubiquitination, respectively, and thereby blocking downstream signal transduction (32, 33). Moreover, USP19 can promote TBK1 degradation via the chaperone-mediated autophagy pathway, providing further negative feedback regulation of antiviral immunity (34).

Inflammatory signaling: USP19 inhibits TNF-α- and IL-1β-triggered NF-κB signaling pathway activation by deubiquitinating TAK1 (35). In macrophages, USP19 exerts anti-inflammatory effects by promoting autophagy-mediated ROS clearance to suppress NLRP3 inflammasome activation; simultaneously, it can stabilize free NLRP3 protein not assembled into inflammasomes through deubiquitination, which then binds IRF4 and prevents its p62-mediated selective autophagic degradation, thereby promoting M2 macrophage polarization and exerting anti-inflammatory functions (36). In acute lung injury models, USP19 also alleviates LPS-induced pulmonary inflammation and endothelial cell apoptosis by inhibiting the TAK1-JNK/p38 signaling axis (37). Adaptive immunity: USP19 inhibits Th17 cell differentiation and Th17-driven autoimmune diseases by deubiquitinating RORγt (38).

**Figure 2:**
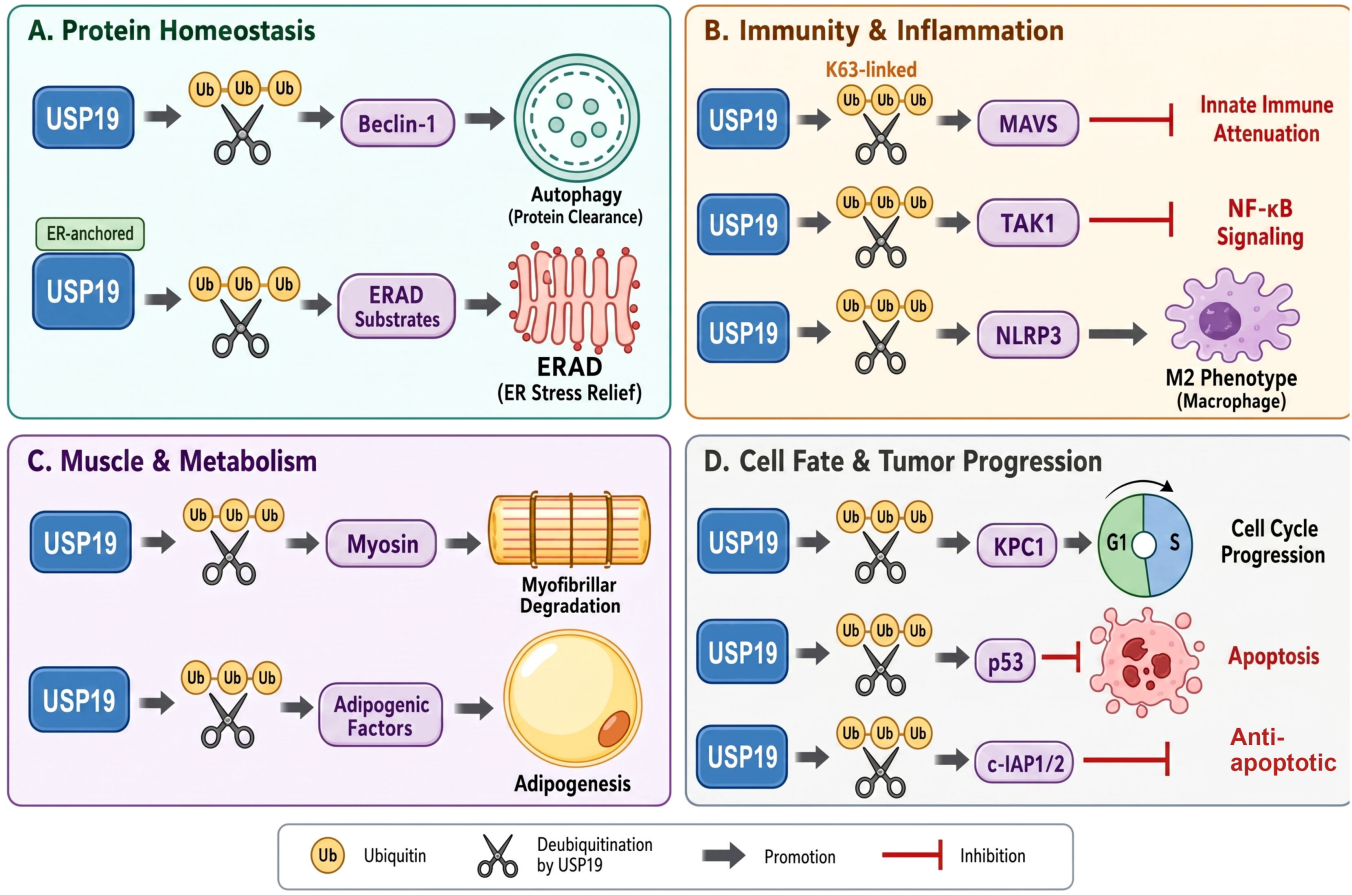
Modular physiological functions and key substrates of USP19. USP19 governs four functional modules: **(A)** protein homeostasis (ERAD, autophagy via Beclin-1); **(B)** immunity and inflammation (MAVS, TAK1, NLRP3-mediated M2 macrophage polarization); **(C)** muscle and metabolism (myofibrillar proteins, adipogenic factors); **(D)** cell fate and tumor progression (KPC1, p53, c-IAP1/2). Ubiquitin linkage types are indicated where experimentally determined.

Cell cycle, proliferation, and death regulation: USP19 exhibits complex functions in this regard. It supports cell cycle progression from G1 to S phase and promotes cell proliferation by stabilizing KPC1 (the ubiquitin ligase for p27Kip1), thereby promoting p27Kip1 degradation (39). In apoptosis regulation, USP19 inhibits Caspase cascade reactions and cell apoptosis by deubiquitinating and stabilizing c-IAP1 and c-IAP2 (40). USP19 also negatively regulates the p53 tumor suppressor protein: it directly interacts with p53 and promotes its ubiquitination, accelerating p53 degradation and thereby suppressing its pro-apoptotic function (41). Furthermore, in an Alzheimer’s disease cell model, USP19 promotes Aβ-induced mitochondrial damage and ferroptosis by stabilizing NOX4 protein (42). Conversely, in a liver ischemia-reperfusion injury model, USP19 exerts a protective effect by deubiquitinating and stabilizing SLC7A11 to inhibit hepatocyte ferroptosis (43) (**Figure 2**).

### 2.4 Functional position within the deubiquitinase network

USP19 operates within a densely interconnected deubiquitinase landscape. Although it possesses unique structural features—such as the ER-anchored splice variant containing a C-terminal transmembrane domain—its functional network overlaps significantly with USP7, USP10, and other family members.

In p53 pathway regulation, USP19 binds DnaJC7 to interfere with the p53-MDM2 interaction and thereby affects p53 stability, whereas USP7 and USP10 bind p53 directly in a manner regulated by PHD3-mediated p53 hydroxylation; together these deubiquitinases constitute critical nodes in the dynamic equilibrium of p53 ubiquitination (44). Notably, USP19 has been reported to promote p53 ubiquitination and shorten its half-life, suggesting that it may form an antagonistic or cooperative relationship with USP7 and USP10 through indirect mechanisms such as MDM2 or cofactor modulation, rather than through simple functional redundancy.

At the substrate level, USP19 stabilizes c-Myc, SLC7A11, and phenylalanine hydroxylase, while USP7 similarly targets N-Myc and other oncogenes (45), and USP10 participates in KLF4 regulation. These patterns imply that different USPs may achieve fine-tuned control over shared signaling axes—such as the MYC family—through competitive binding or spatiotemporal expression differences. In cellular stress responses, USP19 attenuates type I interferon signaling by deubiquitinating MAVS, whereas USP15 has also been implicated in immune and inflammatory pathways (46).

Structurally, USP family members including USP19 rely on catalytic and auxiliary domains for substrate recognition (47). However, the transmembrane domain specific to USP19 may confer subcellular localization specificity relevant to ER and lysosome-associated secretory regulation, whereas USP7 requires C-terminal Ubl domain folding for activation and USP12/USP46 depends on UAF1/WDR20 co-activation (48). These differences indicate that functional specialization arises from subtle structural variations and distinct interaction partners.

## 3 Molecular mechanisms of USP19 in disease

### 3.1 Protein homeostasis, ERAD, and proteotoxic stress responses

USP19 participates in protein quality control through multiple pathways. As an ER-resident deubiquitinase, it recognizes and stabilizes specific ERAD substrates, preventing the accumulation of misfolded proteins. Through a catalytic activity-independent mechanism, it promotes the unconventional secretion of misfolded cytosolic proteins, alleviating proteasome dysfunction. Additionally, by stabilizing Beclin-1 and regulating p62 ubiquitination, USP19 modulates macroautophagy and lysosomal exocytosis. In disease contexts, USP19 determines cell fate by stabilizing specific substrates: NOX4 in Alzheimer’s disease and SLC7A11 in hepatic ischemia-reperfusion injury.

#### 3.1.1 Alzheimer’s disease

Alzheimer’s disease (AD) is the most common neurodegenerative disease, characterized by progressive memory loss and cognitive dysfunction; its core pathological changes include intracerebral β-amyloid (Aβ) deposition and neurofibrillary tangle formation (49). Mitochondrial dysfunction is considered one of the key driving factors in AD pathogenesis; excessive Aβ accumulation can lead to increased mitochondrial fission, decreased fusion, and reduced membrane potential (50). Ferroptosis plays an important role in AD occurrence and development (51). NOX4 is a member of the NADPH oxidase family that mediates oxidative stress and ferroptosis by generating large amounts of ROS, and is widely expressed in the brain (52, 53).

In an Aβ_1-40_-induced SH-SY5Y cellular AD model, USP19 was identified as the key deubiquitinase regulating NOX4 protein stability through screening of 40 USP family genes (42). Co-immunoprecipitation experiments confirmed direct interaction between USP19 and NOX4 protein, and USP19 knockdown significantly increased NOX4 polyubiquitination levels, indicating that USP19 maintains NOX4 protein stability through deubiquitinase activity. Aβ_1-40_ treatment decreased SH-SY5Y cell viability, increased apoptosis, elevated MDA and intracellular Fe²^+^ content, reduced GSH/GSSG ratio, and caused mitochondrial dysfunction, manifested as decreased mitochondrial membrane potential, downregulated fusion proteins Mfn1 and Mfn2, and upregulated fission protein Drp1 (42). USP19 knockdown significantly improved all the above indicators: increased cell viability, reduced apoptosis, decreased MDA and Fe²^+^ content, restored GSH/GSSG ratio, restored mitochondrial membrane potential, upregulated Mfn1/Mfn2, and downregulated Drp1 (42).

#### 3.1.2 Ischemia-reperfusion injury in liver transplantation

Liver transplantation is the standard treatment for end-stage liver disease; however, in conventional liver transplantation, cold ischemia of the graft and subsequent reperfusion inevitably lead to ischemia-reperfusion injury (IRI), causing serious complications such as early graft dysfunction and primary non-function after transplantation (54, 55). Ferroptosis is a form of regulated necrosis driven by lethal lipid peroxidation, with morphological, genetic, and biochemical characteristics distinct from apoptosis, necroptosis, and pyroptosis (56, 57). The SLC7A11-GSH-GPX4 axis is the core pathway inhibiting ferroptosis: SLC7A11 mediates cystine uptake for glutathione synthesis (58), while GPX4 utilizes GSH to reduce highly toxic phospholipid hydroperoxides to non-toxic lipid alcohols (59). Studies have shown that ferroptosis participates in the pathogenesis of hepatic IRI (56, 60).

A study first discovered through proteomic comparison that ferroptosis is the most significantly differentially regulated programmed cell death pathway between ischemia-free liver transplantation (IFLT) and conventional liver transplantation; in IFLT grafts, ferroptosis was significantly suppressed, manifested as reduced mitochondrial shrinkage, decreased lipid peroxidation product MDA levels, elevated antioxidant enzyme SOD activity, and upregulated SLC7A11, GSH, and GPX4 protein levels (43). Further deubiquitinase screening confirmed that USP19 is the most significantly upregulated deubiquitinase in IFLT grafts, and its expression level is significantly positively correlated with the degree of ferroptosis inhibition and the extent of liver injury alleviation. Mechanistically, it was proven that USP19 can directly bind to SLC7A11 protein and specifically remove K63-linked polyubiquitin chains on lysine residue 500 (K500) (43). By blocking this ubiquitination modification, USP19 delays SLC7A11 degradation via the autophagy-lysosome pathway, thereby maintaining its protein stability. The protective effect of USP19 on SLC7A11 is entirely dependent on its intact deubiquitinase activity, as catalytically inactive mutants (C506S and H1157A) cannot stabilize SLC7A11. *In vivo* rescue experiments, knockdown of Slc7a11 in mouse liver via AAV-shRNA completely eliminated the protective effect of USP19 overexpression against hepatic IRI (43).

Current evidence linking USP19 to protein homeostasis in disease is limited to specific cellular and animal models. The AD findings derive exclusively from Aβ_1-40_-stimulated SH-SY5Y neuroblastoma cells, without validation in primary neurons, brain organoids, or transgenic mouse models. The hepatic IRI data rely on proteomic comparisons between IFLT and conventional grafts with limited sample sizes, and the AAV-shRNA rescue experiments were performed only in mice. Whether these protein-stabilizing functions of USP19 operate in human tissues under native pathological conditions remains unverified.

### 3.2 Immune regulation, inflammation, and tumor immune microenvironment

USP19 functions as a pleiotropic negative regulator of immune and inflammatory signaling. It directly removes K63-linked ubiquitin chains from MAVS to suppress aggregation and downstream type I interferon activation. It deubiquitinates TRAF3 and TRIF to block innate immune signal transduction. In inflammatory contexts, USP19 inhibits TNF-α- and IL-1β-triggered NF-κB activation by deubiquitinating TAK1. In macrophages, it promotes autophagy-mediated ROS clearance to suppress NLRP3 inflammasome activation while stabilizing free NLRP3 to promote M2 polarization. These immunomodulatory functions position USP19 at the intersection of sterile inflammation, antiviral defense, and tumor immune evasion.

#### 3.2.1 Colorectal cancer

Colorectal cancer (CRC) is the third most common malignancy worldwide, characterized by a highly immunosuppressive tumor microenvironment that limits the efficacy of immunotherapy (61, 62). PD-L1 is a key immune checkpoint molecule; when it binds to PD-1 on T cells at the tumor cell surface, it inhibits T cell activity and mediates tumor immune evasion (63, 64). PD-L1 protein stability is precisely regulated by ubiquitination modifications, and multiple deubiquitinases are known to stabilize PD-L1 levels by removing its ubiquitin chains (65–67).

Through CRISPR/Cas9 sgRNA library screening and activated T cell co-culture experiments, USP19 was identified as a key molecule regulating T cell-mediated anti-tumor immunity (68). USP19 knockout significantly enhanced the sensitivity of CRC cells to T cell killing, manifested as decreased cell viability, increased apoptosis, elevated Cleaved Caspase-3 levels, and significantly increased secretion of IFN-γ and Granzyme B by CD8^+^ T cells in the co-culture system; conversely, USP19 overexpression inhibited T cell killing effects (68). In immunocompetent mouse models, USP19 deficiency reduced MC38 xenograft tumor growth and increased the infiltration of activated CD8^+^ T cells, PD-1, and GzmB in tumors; however, USP19 deficiency did not affect tumor growth in immunodeficient nude mice, indicating that its effect depends on the adaptive immune system; these findings were also validated in patient-derived CRC organoids (68).

Mechanistically, USP19 directly binds to the intracellular domain of PD-L1 through its transmembrane domain, and utilizes its deubiquitinase activity to specifically remove K48-linked polyubiquitin chains from PD-L1, blocking its proteasomal degradation pathway and thereby stabilizing PD-L1 protein; the catalytically inactive mutant C506S cannot stabilize PD-L1 (68). Clinical sample analysis showed that USP19 is highly expressed in CRC tissues, positively correlates with PD-L1 levels, and is significantly associated with higher tumor grade, poorer differentiation, and TP53 mutation; *in vivo* treatment experiments demonstrated that USP19 deficiency combined with αPD-L1 antibody therapy synergistically inhibited CRC progression (68). Additionally, USP19 can enhance the pro-carcinogenic function of malic enzyme 1 (ME1) in CRC by deubiquitinating and stabilizing ME1, and this process depends on phosphorylation modification of ME1 by ERK2 (16).

#### 3.2.2 Pathological cardiac hypertrophy

Pathological cardiac hypertrophy is an initial adaptive response of the heart to pressure overload or neurohumoral stimulation, but its persistence leads to ventricular remodeling, fibrosis, and even heart failure (69, 70). TAK1 is a member of the MAPKKK family that responds to various stress stimuli and activates downstream p38 and JNK1/2 signaling pathways, participating in the development of cardiac hypertrophy (71, 72). On the other hand, p38 and JNK1/2, as stress-activated MAPKs, have been confirmed to induce cardiomyocyte hypertrophy and cardiac dysfunction when excessively activated (73, 74).

In transverse aortic constriction (TAC)-induced hypertrophic mouse hearts and phenylephrine (PE)-stimulated neonatal rat cardiomyocytes, USP19 protein levels were significantly elevated; this mechanism is partially attributable to transcriptional downregulation of the E3 ubiquitin ligase SIAH2, leading to reduced ubiquitination-mediated degradation of USP19 (1, 15). USP19 knockout mice exhibited more severe cardiac hypertrophy, fibrosis, dysfunction, and inflammatory responses than wild-type mice after TAC surgery, specifically manifested as increased heart weight/body weight ratio and lung weight/body weight ratio, decreased left ventricular ejection fraction and fractional shortening, and significant upregulation of hypertrophic markers such as ANP, BNP, and Myh7, as well as fibrotic markers such as collagen I/III and CTGF (3). The study also found that NF-κB signaling pathway and downstream pro-inflammatory factors IL-1β and TNF-α expression were enhanced after USP19 deletion.

Mechanistic studies revealed that USP19 negatively regulates downstream p38 and JNK1/2 signal transduction by inhibiting TAK1 phosphorylation, without affecting the ERK pathway (35). Treatment of USP19-knockdown cardiomyocytes with the TAK1-specific inhibitor NG25 completely eliminated the p38/JNK1/2 phosphorylation elevation and cellular hypertrophic phenotypes caused by USP19 deficiency (3). Furthermore, the TAK1-JNK1/2 and TAK1-p38 axes also participate in mediating the production of pro-fibrotic factors such as CTGF during cardiac fibrotic responses (75).

#### 3.2.3 Acute lung injury

Acute lung injury (ALI) and its severe form, acute respiratory distress syndrome, are life-threatening clinical syndromes characterized by diffuse alveolar-capillary damage, increased permeability, pulmonary edema, and refractory hypoxemia, with a mortality rate as high as approximately 40%; Gram-negative bacterial endotoxin lipopolysaccharide (LPS) is one of the most common causative factors (76, 77). LPS activates TLR4 signaling, triggering downstream NF-κB and MAPK pathways, inducing massive production of pro-inflammatory cytokines, thereby causing inflammatory cascade reactions and endothelial barrier damage (78, 79).

In LPS-stimulated mouse lung tissues and human pulmonary microvascular endothelial cells (HULEC-5a), USP19 mRNA and protein expression levels were significantly decreased; USP19 knockout mice exhibited more severe lung tissue damage, alveolar hemorrhage, inflammatory cell infiltration, increased pulmonary vascular permeability, and pulmonary edema under LPS stimulation (37). Concurrently, USP19 deficiency promoted LPS-induced pulmonary inflammatory responses, manifested as significantly elevated levels of TNF-α, IL-6, and IL-1β in bronchoalveolar lavage fluid, and increased neutrophil and macrophage infiltration; regarding apoptosis, upregulation of pro-apoptotic molecule Bax and downregulation of anti-apoptotic molecule Bcl2 were also exacerbated after USP19 deficiency (37). *In vitro* experiments showed that USP19 overexpression alleviated LPS-induced endothelial cell damage, inflammatory factor expression, and apoptosis, whereas USP19 knockdown aggravated the above phenotypes. Mechanistically, USP19 negatively regulates downstream JNK and p38 signaling pathway activation by inhibiting TAK1 phosphorylation, and the TAK1-specific inhibitor iTAK1 effectively reversed the pro-inflammatory and pro-apoptotic effects caused by USP19 knockdown (37).

#### 3.2.4 USP19 in the tumor immune microenvironment

USP19 shapes the tumor immune microenvironment through multiple convergent pathways. In tumor cells, USP19 stabilizes PD-L1 to suppress CD8^+^ T cell cytotoxicity (68). In innate immune cells, USP19 deubiquitinates MAVS to attenuate type I interferon production and antiviral surveillance (14), and removes K63- and K27-linked chains from TRAF3 and TRIF respectively to block downstream signal transduction (32, 33). In macrophages, USP19 stabilizes NLRP3 to promote IRF4-dependent M2 polarization, fostering an immunosuppressive microenvironment (36). It also suppresses Th17 differentiation by deubiquitinating RORγt (38). These immunomodulatory functions suggest that USP19 may contribute to a “cold tumor” phenotype in microsatellite-stable solid tumors, though direct clinical evidence linking USP19 expression to immune infiltration scores or checkpoint inhibitor response rates is currently lacking.

**Figure 3:**
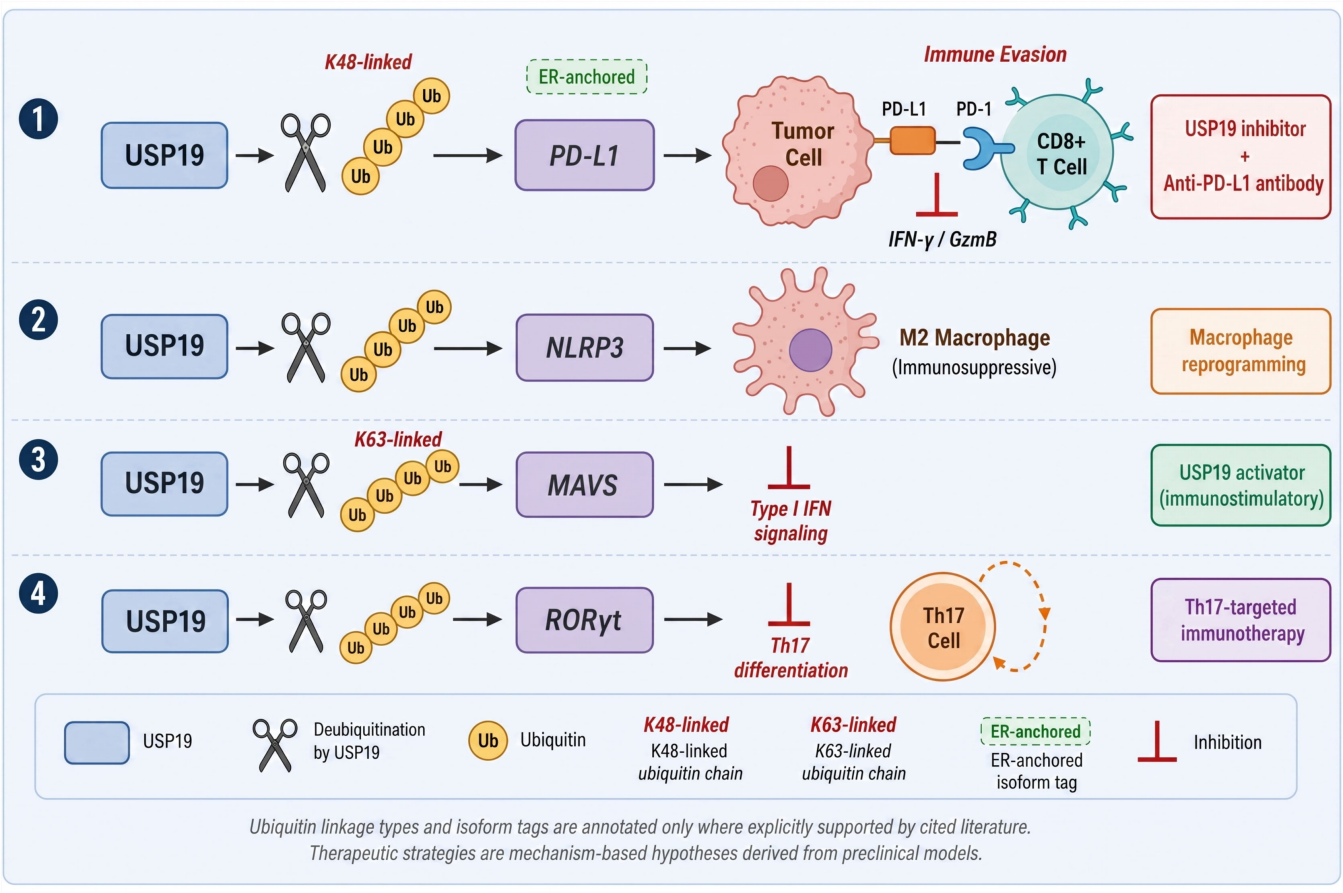
Immunomodulatory mechanisms of USP19 in the tumor microenvironment. Four convergent pathways are depicted: (1) ER-anchored USP19 removes K48-linked ubiquitin chains from PD-L1 to block its proteasomal degradation, thereby suppressing CD8^+^ T cell cytotoxicity; (2) USP19 stabilizes NLRP3 to promote IRF4-dependent M2 macrophage polarization; (3) USP19 deubiquitinates MAVS (K63-linked) to attenuate type I interferon signaling; (4) USP19 deubiquitinates RORγt to inhibit Th17 differentiation. Therapeutic hypotheses are indicated.

The immunomodulatory mechanisms of USP19 are supported primarily by genetic knockout and overexpression models in mice and cell lines. The PD-L1 immune evasion data in CRC rely on MC38 allografts and patient-derived organoids, but no clinical cohort has validated the correlation between USP19 and PD-L1 protein levels in human CRC specimens using standardized immunohistochemistry protocols. The cardioprotective and pulmonary protective effects of USP19 are derived exclusively from Siah2 transcriptional downregulation or gene knockout models, without pharmacological activator validation. The tumor immune microenvironment integration remains a theoretical construct: whether USP19-mediated MAVS attenuation directly suppresses anti-tumor immunity *in vivo*, or whether USP19-driven M2 macrophage polarization operates in human tumor stroma, has not been experimentally demonstrated (**Figure 3**).

### 3.3 Cell proliferation, survival, and DNA damage responses

USP19 regulates cell fate decisions by controlling the stability of key proteins involved in cell cycle progression, apoptosis inhibition, and DNA damage repair. It promotes G1/S transition by stabilizing KPC1, the ubiquitin ligase for p27Kip1. It inhibits apoptosis through c-IAP1/2 stabilization. In cancer contexts, USP19 promotes malignant survival by accelerating p53 degradation, stabilizing oncogenic c-Myc and YAP, and enhancing DNA repair capacity through MGMT stabilization. These functions collectively position USP19 as a pro-survival factor in multiple malignancies.

#### 3.3.1 Clear cell renal cell carcinoma

Renal cell carcinoma (RCC) is one of the most common malignancies of the urinary system, with approximately 430,000 new cases and 180,000 deaths globally in 2020 (56). Clear cell renal cell carcinoma (ccRCC) is the predominant histological subtype, accounting for approximately 80% of all cases (80, 81). Although surgical resection is the most effective treatment for ccRCC, approximately 25% of patients still develop distant metastasis after radical nephrectomy (82), and the 5-year survival rate for metastatic patients is only 5–10% (83).

Analyses of TCGA and GEO databases have revealed that USP19 mRNA levels are significantly lower in ccRCC tumor tissues than in normal renal tissues, and that USP19 copy number is also significantly reduced in ccRCC, suggesting that the downregulation may originate from copy number loss (10). Functionally, overexpression of USP19 significantly inhibited the proliferation and migration of Caki-1 cells, whereas USP19 knockdown produced the opposite effects. At the molecular level, USP19 overexpression downregulated proliferation markers PCNA and cyclin D1, upregulated the cycle inhibitor p27, and decreased the expression of migration markers MMP2 and MMP9 (10). PCNA and cyclin D1 participate in cell cycle regulation, p27 is a cyclin-dependent kinase inhibitor, and MMP2 and MMP9 are classical markers of tumor cell migration (84–87).

The ERK/MAPK signaling pathway is involved in the regulation of proliferation and migration in various cancers (88, 89). Studies have found that USP19 overexpression reduces ERK phosphorylation levels, whereas USP19 knockdown significantly increases ERK phosphorylation (10). Concurrent use of the ERK pathway inhibitor U0126 reversed the enhanced cell proliferation and migration abilities caused by USP19 knockdown; *in vivo* experiments also confirmed that USP19 knockdown significantly promoted xenograft tumor growth in nude mice, with elevated p-ERK levels in tumor tissues (10).

#### 3.3.2 Cervical cancer

Cervical cancer is the fourth most common malignancy in women worldwide, and its occurrence is closely related to human papillomavirus (HPV) infection (90). p53 is one of the most important tumor suppressor proteins; it inhibits cell cycle progression and induces apoptosis under cellular stress, and its protein levels are precisely regulated by multiple E3 ligases and deubiquitinases (91, 92). Some deubiquitinases such as USP7 and USP10 can stabilize p53 through deubiquitination (93, 94), whereas others such as USP4 and USP15 indirectly promote p53 degradation by stabilizing E3 ligases such as MDM2 (95, 96). In HeLa cervical cancer cells, USP19 was identified as a negative regulator of p53; USP19 directly interacts with p53 protein and promotes p53 polyubiquitination through its deubiquitinase activity, shortening its protein half-life and thereby reducing p53 protein levels (41). CRISPR/Cas9-mediated USP19 knockout significantly upregulated p53 protein levels in HeLa cells; functionally, USP19 knockout significantly inhibited HeLa cell anchorage-independent colony formation, migration, and invasion abilities, whereas USP19 overexpression rescued these malignant phenotypes (41).

#### 3.3.3 Diffuse large B-cell lymphoma

Diffuse large B-cell lymphoma (DLBCL) is the most common subtype of non-Hodgkin lymphoma, accounting for approximately 30% of all cases; about one-third of refractory patients have an extremely poor prognosis (97, 98). PARK7 (also known as DJ-1) is a member of the peptidase C56 family; as a multifunctional protein it participates in oxidative stress responses and cell survival regulation, and has been found to exert pro-carcinogenic functions in various cancers (99, 100).

GEPIA database analysis shows that USP19 is significantly overexpressed in DLBCL tissues, and its high expression is associated with poorer overall survival in patients (101). In two DLBCL cell lines (DB and SUDHL4), USP19 knockdown significantly inhibited cell viability, colony formation ability, and G1/S cell cycle progression, while downregulating cyclin D1 and upregulating p27Kip1 expression; conversely, USP19 overexpression promoted cell proliferation and cell cycle progression; *in vivo* nude mouse xenograft experiments showed that USP19 knockdown significantly reduced tumor volume and weight, and decreased Ki67 staining while increasing p27Kip1 expression (101).

Through Co-IP coupled with liquid chromatography-mass spectrometry analysis, PARK7 was identified as a USP19-interacting protein; USP19 directly binds to PARK7 and delays PARK7 degradation via the proteasome pathway through deubiquitination, thereby stabilizing PARK7 protein (101). Reports have indicated that PARK7 can promote cancer cell survival by activating the Akt signaling pathway (102). Functional rescue experiments confirmed that PARK7 overexpression reversed the proliferation inhibition and cell cycle arrest in DLBCL cells caused by USP19 knockdown (101).

#### 3.3.4 Glioblastoma

Glioblastoma (GBM) is the most common and aggressive primary brain tumor in adults, with a median survival of approximately 15 months (103, 104). Temozolomide (TMZ) is the standard first-line chemotherapeutic agent; however, primary resistance is common. O^6^-methylguanine-DNA methyltransferase (MGMT) repairs TMZ-induced DNA alkylation damage, and its expression level is a major determinant of TMZ sensitivity (105–107). The MGMT protein is degraded via the ubiquitin-proteasome pathway after DNA repair is completed, but its deubiquitination regulation mechanism remained unclear previously (108).

In T98G glioblastoma cells, which express high levels of MGMT, USP19 knockdown significantly reduced MGMT protein levels, increased TMZ-induced DNA damage (γH2AX foci), and enhanced TMZ cytotoxicity and apoptosis. In contrast, in MGMT-negative U251 and U87 cell lines, USP19 knockdown did not affect TMZ sensitivity; *in vivo* experiments confirmed that T98G xenografts with USP19 knockdown were more sensitive to TMZ treatment (109). Concurrently, studies also found that in GBM patient clinical samples, USP19 and MGMT protein levels were positively correlated, and patients with USP19 genetic alterations had poorer prognosis.

DNA Damage Response and Genomic Stability. Beyond MGMT-mediated alkylating resistance, whether USP19 modulates other DNA damage response components such as ATM, ATR, or BRCA1 remains unexplored. USP19 promotes G1/S transition by stabilizing KPC1, the ubiquitin ligase for p27Kip1 (39); this accelerated cell cycle entry may increase replication stress and genomic instability under oncogenic pressure, though direct evidence linking USP19 to DNA double-strand break repair or chromosomal integrity is currently lacking.

The evidence for USP19 as a pro-survival factor in this module derives predominantly from retrospective database analyses, single cell line models, and xenograft experiments. The ccRCC findings are limited to Caki-1 cells and nude mice, without validation in patient-derived models or humanized immune systems. The cervical cancer p53 mechanism was demonstrated exclusively in HeLa cells, which carry HPV E6 that independently targets p53; whether this mechanism operates in HPV-negative cervical cancer or primary tumor tissues is unknown. The DLBCL data rely on GEPIA correlation and two cell lines, without clinical sample validation of the USP19-PARK7 interaction. The GBM TMZ resistance mechanism was validated in T98G cells but not in MGMT-negative models, and the proposed link between KPC1-mediated cell cycle control and genomic instability remains theoretical.

### 3.4 Tumor invasion, metabolic reprogramming, and microenvironment remodeling

USP19 drives malignant progression through distinct mechanisms that converge on tissue invasion, metabolic adaptation, and microenvironment modification. In gastric cancer and hepatocellular carcinoma, it upregulates matrix metalloproteinases to degrade extracellular matrix barriers. In triple-negative breast cancer, the ER-anchored isoform stabilizes LRP6 to hyperactivate Wnt signaling and promote distant metastasis. In hepatocellular carcinoma, USP19 sustains aerobic glycolysis and metastatic capacity through c-Myc and YAP stabilization. These functions highlight the isoform- and context-specific nature of USP19-mediated tumor progression.

#### 3.4.1 Gastric cancer

Gastric cancer is the fifth leading cause of cancer-related death worldwide. Most patients are diagnosed at an advanced stage with metastasis, and the 5-year survival rate is below 10% (61, 110, 111). Matrix metalloproteinases MMP2 and MMP9 promote tumor invasion and metastasis by degrading extracellular matrix components, and their expression is significantly elevated during the invasive progression of gastric cancer (112).

Functionally, USP19 knockdown in SGC7901 gastric cancer cells inhibited cell proliferation and colony formation, and increased the levels of apoptosis-related protein Cleaved Caspase-3; conversely, GES1 immortalized gastric epithelial cells overexpressing USP19 acquired enhanced proliferation, colony formation capacity, and anti-apoptotic characteristics (113). Regarding migration and invasion, USP19 knockdown reduced the invasion and migration abilities of SGC7901 cells, while simultaneously decreasing MMP2 and MMP9 protein expression and gelatinase activity; conversely, USP19 overexpression enhanced the invasion and migration abilities of GES1 cells and upregulated MMP2/MMP9 expression and activity (113). Other USP family members are also known to participate in regulating MMP expression and cell migration; for example, USP6 can directly induce MMP9 transcription by activating NF-κB (114), and USP22 knockdown inhibits osteosarcoma metastasis via the PI3K/Akt pathway (115). *In vivo* experiments confirmed that USP19 knockdown inhibited xenograft tumor growth in nude mice, with decreased MMP2 and MMP9 expression and elevated Cleaved Caspase-3 expression in tumor tissues (113).

#### 3.4.2 Hepatocellular carcinoma

Hepatocellular carcinoma (HCC) is one of the most common malignancies worldwide, accounting for approximately 75–85% of liver cancer cases, and has a poor prognosis (116). c-Myc is a classic oncoprotein overexpressed in various human cancers that can drive tumor development by reprogramming cellular metabolism (117–119); E2F1 is a member of the E2F transcription factor family that plays important functions in regulating proliferation and apoptosis in HCC (119).

Studies have confirmed that E2F1 can directly bind to the USP19 promoter region and activate its transcription in HCC, leading to high USP19 expression in HCC tissues and cells (11). At the molecular mechanism level, USP19 directly binds to c-Myc through its deubiquitinase activity, removing polyubiquitin chains from c-Myc to maintain its protein stability, thereby delaying c-Myc degradation via the ubiquitin-proteasome pathway (11). Functionally, c-Myc silencing inhibited HCC cell proliferation, migration, invasion, sphere formation capacity, and glycolysis—the latter being a core feature of the c-Myc-driven Warburg effect—whereas USP19 maintained these malignant phenotypes by stabilizing c-Myc protein (120). Rescue experiments confirmed that c-Myc overexpression reversed the anti-tumor effects caused by USP19 knockdown; *in vivo* experiments showed that USP19 knockdown inhibited xenograft tumor growth and lung metastasis nodule formation in nude mice by downregulating c-Myc (11). Additionally, USP19 can promote HCC progression by deubiquitinating and stabilizing YAP protein (121).

#### 3.4.3 Triple-negative breast cancer

Triple-negative breast cancer (TNBC) is the breast cancer subtype with the poorest prognosis, lacking expression of estrogen receptor, progesterone receptor, and HER2, and therefore lacking effective targeted therapeutic options (122, 123). Abnormal activation of the Wnt signaling pathway plays an important role in breast cancer development and progression; this pathway drives pro-invasive transcriptional programs by stabilizing β-catenin (124).

In TNBC cells, USP19 was confirmed to positively regulate cell migration and invasion abilities, and this promoting effect depends on its intact deubiquitinase activity and ER anchoring—neither the catalytically inactive mutant (C506S) nor the transmembrane domain-deleted isoform possesses this function (19). Mechanistically, USP19 enhances Wnt signaling pathway activity by deubiquitinating and stabilizing the Wnt co-receptor LRP6 protein (19). LRP6 is a key receptor for Wnt signaling, and its protein stability directly determines Wnt signaling intensity (124). Retrospective analysis of clinical samples from early-stage breast cancer patients indicated that high USP19 expression is significantly associated with shorter distant metastasis-free survival, suggesting its potential as a prognostic marker (19). Additionally, in TNBC, USP19 can also regulate apoptotic calcium release and ER stress by deubiquitinating and stabilizing BAG6 protein (125).

The invasion and metabolic mechanisms attributed to USP19 in this module are supported by *in vitro* cell line experiments and retrospective clinical correlations. The gastric cancer MMP2/9 data were generated in SGC7901 and GES1 cells, but the direct upstream mechanism linking USP19 to MMP expression—whether through transcriptional co-regulation or purely post-translational stabilization—remains undefined. The HCC c-Myc/YAP mechanisms were validated in xenograft models, yet the cooperative versus independent contributions of these two substrates to glycolysis and metastasis were not dissected. The TNBC findings are limited to early-stage patient cohorts with small sample sizes, and the requirement for ER anchoring in LRP6 stabilization raises critical questions about isoform-selective therapeutic targeting that have not been addressed experimentally.

### 3.5 Synthesis: context-dependent functional duality and the immuno-regulatory hub

The divergent pathological roles of USP19—oncogenic in gastric cancer, hepatocellular carcinoma, and diffuse large B-cell lymphoma, yet tumor-suppressive in clear cell renal cell carcinoma and protective in acute lung injury and pathological cardiac hypertrophy—cannot be explained by a single mechanism. Instead, they arise from the intersection of four contextual determinants.

First, substrate availability differs across tissues. In ccRCC, USP19 negatively regulates ERK phosphorylation, suppressing proliferation and migration; in gastric cancer, USP19 upregulates MMP2 and MMP9 to promote extracellular matrix degradation. The opposing phenotypes reflect the differential reliance of these malignancies on ERK versus MMP-driven invasion.

Second, isoform distribution creates functional compartmentalization. The ER-anchored isoform promotes TNBC invasion through LRP6 stabilization at the membrane, whereas the cytosolic isoform uc003cvz.3 is downregulated in advanced ccRCC and correlates with poor prognosis. The absence of the transmembrane domain in the latter may restrict its substrate repertoire to cytosolic or nuclear proteins.

Third, upstream transcriptional control sets the abundance threshold. E2F1 drives USP19 overexpression in HCC, creating a pro-tumorigenic environment, whereas 3p21.3 heterozygous deletion in ccRCC causes USP19 loss. The direction of transcriptional dysregulation thus dictates whether USP19 functions as an oncogenic enabler or a tumor suppressor.

Fourth, and most critically, the immune microenvironment acts as a polarity switch. In colorectal cancer, USP19 stabilizes PD-L1 to suppress CD8^+^ T cell cytotoxicity and promotes M2 macrophage polarization, fostering immune evasion. In acute lung injury and pathological cardiac hypertrophy, the same USP19-TAK1 axis inhibits p38/JNK-driven inflammation and prevents tissue destruction. The identical molecular interaction produces diametrically opposed organismal outcomes depending on whether the tissue context is a tumor exploiting immune tolerance or a sterile inflammatory injury requiring resolution.

**Figure 4:**
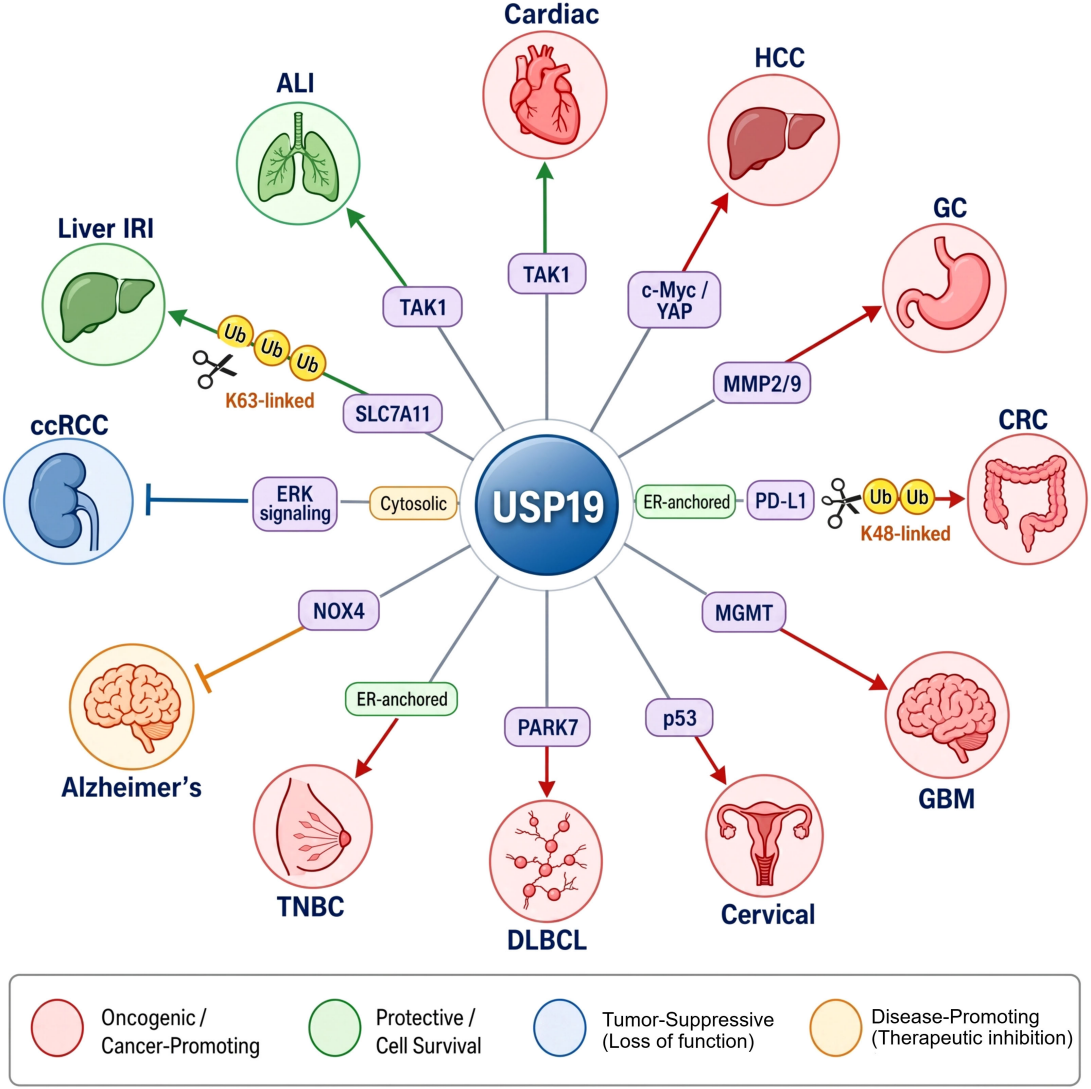
Panoramic landscape of USP19-mediated pathologies and clinical implications. Context-dependent roles of USP19 in neoplastic (HCC, GC, CRC, GBM, DLBCL, TNBC, cervical cancer), tumor-suppressive (ccRCC), and non-neoplastic diseases (Alzheimer’s disease, pathological cardiac hypertrophy, ALI, hepatic IRI). Color coding denotes functional polarity (oncogenic/cancer-promoting, protective/cell survival, tumor-suppressive, neurodegenerative/pathology inhibition). Isoform distribution (ER-anchored vs. cytosolic) and ubiquitin linkage types (K48-linked, K63-linked) are annotated.

These contextual layers are not mutually exclusive; they likely operate in combination. However, current evidence does not permit quantitative prediction of which polarity will dominate in a given tissue. Single-cell transcriptomic analyses of USP19 isoform expression across tumor and stromal compartments, together with conditional isoform-specific knockout models, are needed to establish predictive rules. Without such data, the context-dependent duality of USP19 remains an empirical observation rather than a mechanistically resolved principle (**Figure 4**).

## 4 Clinical translation of USP19

Current USP19 research has advanced from basic mechanistic exploration to clinical translational validation. However, to transform existing retrospective association findings into evidence-based guidance for clinical decision-making, it is necessary to move beyond simple “expression difference—clinical parameter” descriptive models and instead construct an actionable framework centered on patient stratification, treatment selection, and dynamic monitoring. The following sections outline the clinical translational pathway of USP19 and its existing evidence base from two dimensions: biomarker application and therapeutic target development.

### 4.1 Biomarkers

#### 4.1.1 Diagnosis and prognostic stratification

USP19 expression changes exhibit significant directional heterogeneity across different diseases, and this bidirectional prognostic pattern suggests its potential as a tissue-specific prognostic stratification marker. In ccRCC, USP19 mRNA and the main cytoplasmic isoform uc003cvz.3 are significantly downregulated in tumor tissues due to copy number loss, and this low expression state is significantly associated with higher tumor stage, pathological grade, and poorer overall survival (OS) and disease-free survival (DFS) (8, 10). This finding suggests that after radical nephrectomy, low USP19 expression could identify patients at elevated postoperative recurrence risk, used to refine intermediate-risk populations not adequately identified by conventional clinical staging. Conversely, in gastric cancer, HCC, and DLBCL, high USP19 expression consistently predicts poor prognosis. An immunohistochemical study including 212 gastric cancer specimens confirmed that the USP19 protein overexpression rate reached 66.5%, and it serves as an independent prognostic predictor for gastric cancer patients (113); TCGA and GEPIA database analyses also support that high USP19 expression in HCC and DLBCL is significantly associated with poorer OS (11, 101). In early-stage triple-negative breast cancer (TNBC) patients, high USP19 expression is similarly associated with shorter distant metastasis-free survival (19). Therefore, the prognostic value of USP19 has clear disease dependency: as a “protective deletion” marker in ccRCC, and as an “oncogenic overexpression” marker in gastric cancer, HCC, DLBCL, and TNBC. In future multi-cancer prospective cohorts, USP19 expression levels could be integrated into existing clinical staging systems as auxiliary parameters for risk re-stratification, to identify high-risk patient subgroups requiring intensified adjuvant therapy or close follow-up.

#### 4.1.2 Treatment response prediction

Compared with relatively abundant prognostic data, the predictive value of USP19 has not yet been systematically mined, but existing evidence has revealed important clues. In glioblastoma (GBM), USP19 directly confers primary resistance to temozolomide (TMZ) in MGMT-positive tumor cells by deubiquitinating and stabilizing the DNA repair protein MGMT (109). This means that USP19 expression levels are not only related to prognosis but may also predict objective response rates to first-line TMZ chemotherapy; for patients with high USP19 expression and MGMT positivity, clinicians could consider alternative alkylating agent regimens or combination DNA damage repair inhibitors in advance. In colorectal cancer (CRC), USP19 mediates tumor immune evasion by stabilizing the immune checkpoint molecule PD-L1, and its expression level is positively correlated with PD-L1 protein levels (63). Based on this, it is speculated that high USP19 expression may define a class of “cold tumor” microenvironments with stronger immunosuppressive properties; these patients may benefit more from USP19 inhibitors combined with anti-PD-1/PD-L1 immunotherapy, whereas patients with low USP19 expression may already respond well to single-agent immune checkpoint inhibitors. In gastric cancer, database analysis shows that among patients receiving 5-FU adjuvant chemotherapy, those with high USP19 mRNA expression had significantly inferior overall survival compared with those with low expression (113), strongly suggesting that high USP19 expression may predict primary 5-FU resistance, providing a molecular basis for clinical chemotherapy regimen changes or combined targeted interventions.

Furthermore, in HCC, USP19 maintains the tumor glycolytic phenotype (Warburg effect) by stabilizing c-Myc (11), and can further promote malignant progression by stabilizing YAP (121); based on this metabolic regulatory characteristic, whether USP19 expression levels can predict the efficacy of anti-angiogenic drugs or metabolic targeted interventions deserves validation in subsequent clinical studies. USP19 may serve as both a prognostic and a predictive biomarker, helping to identify which patients are sensitive or resistant to specific treatments.

#### 4.1.3 Dynamic monitoring and treatment process management

Biomarkers ultimately serve both static pre-treatment stratification and dynamic treatment monitoring. In the liver transplantation field, USP19 protein levels in ischemia-free liver transplantation (IFLT) grafts are significantly higher than in traditional cold preservation grafts, and its expression level is significantly negatively correlated with postoperative transaminase peak values, Suzuki histological injury scores, and early graft dysfunction incidence (43). This finding makes USP19 a promising objective indicator for assessing the degree of graft ischemia-reperfusion injury, useful for dynamic prediction of early postoperative liver function recovery. Given that USP19 has transmembrane domain isoforms and cytoplasmic isoforms (2, 7), and participates in the unconventional secretion pathway of misfolded proteins (21, 22), future exploration of dynamic changes in USP19 isoforms in serum or plasma as liquid biopsy biomarkers could enable real-time monitoring of tumor burden, treatment response, or organ injury repair status. Although this application remains at the conceptual stage, its molecular characteristics provide a rationale for developing non-invasive dynamic monitoring tools.

#### 4.1.4 Bioinformatic corroboration

In silico analysis of publicly available datasets provides convergent support for USP19’s biomarker potential. In HCC, Gene Set Enrichment Analysis (GSEA) of TCGA RNA-seq data reveals that tumors with high USP19 expression significantly enrich MYC target genes, glycolysis, and mTOR signaling pathways, consistent with USP19-mediated c-Myc stabilization and the Warburg effect (11). Conversely, in ccRCC, low USP19 expression correlates with enrichment of epithelial-mesenchymal transition (EMT) and hypoxia signatures (10). In GBM, USP19-high tumors show positive correlation with DNA repair and MGMT-related gene modules (97). These computational associations, while requiring independent validation in prospective cohorts, demonstrate that USP19 expression aligns with known disease-driving pathways rather than representing a non-specific bystander effect. Multi-omics integration further suggests that USP19 could be incorporated into multi-gene prognostic signatures—combining with MGMT, PD-L1, or c-Myc rather than serving as an isolated biomarker—to improve predictive accuracy across heterogeneous patient populations (**Table 1**).

**Table 1:** USP19 as a candidate biomarker: clinical applications and evidence tiers.

Application tier	Disease	Biomarker type	Expression pattern	Proposed clinical action	Evidence tier	Key evidence	Ref.
Prognostic stratification	Clear cell renal cell carcinoma	Protective loss marker	Low tissue mRNA/cytosolic isoform (uc003cvz.3)	Postoperative risk re-stratification after radical nephrectomy	Retrospective database (TCGA/GEO)	Low expression correlates with advanced stage, high grade, and poor OS/DFS	(8, 10)
	Gastric cancer	Oncogenic overexpression marker	High tissue protein	Early warning of poor prognosis; intensified follow-up	Retrospective cohort (single-center IHC, 212 specimens)	Overexpression rate of 66.5%; independent prognostic factor	(113)
	Hepatocellular carcinoma	Oncogenic overexpression marker	High tissue mRNA/protein	Survival prediction and adjuvant therapy selection	Retrospective database (TCGA/GEPIA)	High expression is associated with poor OS	(11)
	Diffuse large B-cell lymphoma	Oncogenic overexpression marker	High tissue mRNA	Survival prediction and chemotherapy intensity adjustment	Retrospective database (GEPIA)	High expression correlates with poor OS	(101)
	Triple-negative breast cancer	Metastasis prediction marker	High tissue mRNA/protein	Distant metastasis-free survival prediction	Retrospective cohort (early-stage patients)	High expression is associated with shortened DMFS	(19)
Therapeutic prediction	Glioblastoma	Chemoresistance predictor	High tissue protein	TMZ efficacy prediction and alternative regimen screening	Preclinical (cellular/xenograft models)	Positively correlated with MGMT; mediates TMZ resistance	(109)
	Colorectal cancer	Immunotherapy response predictor	High tissue mRNA/protein	Strategy screening for anti-PD-1/PD-L1 combination therapy	Preclinical (cellular/organoid/immunocompetent mouse)	Positively correlated with PD-L1; USP19 inhibition enhances therapeutic efficacy	(68)
	Gastric cancer	Chemotherapy response predictor	High tissue mRNA	Efficacy prediction of 5-FU adjuvant chemotherapy	Retrospective database analysis	Patients with high expression exhibit inferior OS after 5-FU adjuvant chemotherapy	(113)
Dynamic monitoring	Hepatic ischemia-reperfusion injury after liver transplantation	Injury evaluation marker	Upregulated protein in allograft	Early postoperative allograft function assessment	Preclinical (proteomic comparison; animal models)	Negatively correlated with peak transaminase level and Suzuki histological score	(43)

OS, overall survival; DFS, disease-free survival; DMFS, distant metastasis-free survival; TMZ, temozolomide. All biomarker applications are supported by preclinical or retrospective evidence (Evidence Tier III or below); no prospective clinical trial has validated USP19 as a companion diagnostic. The dynamic monitoring tier currently relies on allograft tissue expression. Serum or plasma isoform detection as a liquid biopsy remains conceptual and requires prospective assay development.

### 4.2 USP19 as a therapeutic target: intervention strategies

USP19 regulates core pathways of various diseases through its deubiquitinase activity, making it an attractive direct drug target. According to disease type and the functional role of USP19 (oncogenic vs. protective), intervention strategies should follow the principle of “inhibition in oncogenic diseases, activation in protective diseases,” and combination regimens should be designed based on specific molecular mechanisms. The following strategies are mechanism-based hypotheses derived from preclinical genetic models; no selective small-molecule USP19 modulator has entered clinical trials.

#### 4.2.1 Inhibition strategies

In tumors such as gastric cancer, HCC, CRC, cervical cancer, DLBCL, and GBM, USP19 exerts oncogenic functions by stabilizing oncoproteins or immune checkpoint molecules. Based on substrate characteristics and signaling pathway properties, inhibition strategies can be divided into three categories: (1) Substrate degradation-type inhibition: In HCC, USP19 stabilizes c-Myc and YAP through deubiquitination, maintaining tumor proliferation, glycolysis, and metastasis (11, 66); in DLBCL, USP19 stabilizes PARK7 to activate Akt pro-survival signaling (102); in cervical cancer, USP19 weakens tumor suppressor function by promoting p53 ubiquitination and degradation (41). For this scenario, USP19 small-molecule inhibitors could prevent p53 degradation, restore its protein stability, and reactivate p53 downstream pro-apoptotic and cell cycle arrest functions. (2) Immune checkpoint modulation-type inhibition: In CRC, USP19 maintains PD-L1 protein levels and mediates immune evasion by removing K48-linked ubiquitin chains to block its proteasomal degradation (63). USP19 inhibitors could downregulate PD-L1 expression, reshape the tumor immune microenvironment, and enhance the infiltration and killing functions of cytotoxic T cells. (3) DNA damage repair-type inhibition: In GBM, USP19 stabilizes MGMT protein, directly antagonizing TMZ-induced DNA alkylation damage repair (109). Inhibiting USP19 could accelerate MGMT degradation, thereby reversing TMZ resistance and restoring tumor cell sensitivity to alkylating agents.

Based on the above mechanisms, combination therapeutic strategies should possess clear molecular synergistic logic. In GBM, USP19 inhibitors combined with TMZ could enhance chemotherapy efficacy by downregulating MGMT (97); in CRC, USP19 inhibitors combined with anti-PD-L1 antibodies could synergistically enhance anti-tumor immunity by promoting PD-L1 degradation (63). These combination regimens follow the design principle of “targeting upstream and downstream nodes of the same resistance axis,” possessing a mechanistic foundation for entering preclinical research.

#### 4.2.2 Activation strategies

In diseases such as pathological cardiac hypertrophy, acute lung injury (ALI), and liver transplantation ischemia-reperfusion injury (IRI), USP19 exerts endogenous protective functions, and its pharmacological activation or expression upregulation has therapeutic potential.

1. Anti-inflammatory and anti-fibrotic activation: In pressure overload-induced cardiac hypertrophy and LPS-induced ALI models, USP19 inhibits excessive activation of downstream p38 and JNK1/2 signaling pathways by deubiquitinating TAK1, thereby alleviating cardiomyocyte hypertrophy, fibrosis, and alveolar endothelial cell apoptosis (1, 37). Activating USP19 could simulate this protective effect. Potential intervention pathways include: screening for small molecules that relieve USP19 N-terminal CS domain intramolecular autoinhibition (mimicking HSP90 activation effects) (4), or developing molecules that interfere with E3 ubiquitin ligase SIAH1/SIAH2-mediated USP19 ubiquitination and degradation to maintain USP19 protein homeostasis (3, 15).
2. Ferroptosis inhibition-type activation: In liver transplantation IRI, USP19 inhibits hepatocyte ferroptosis by deubiquitinating and stabilizing SLC7A11, maintaining glutathione synthesis and GPX4 activity (43). Local overexpression of USP19 through AAV vectors or nano-delivery systems, or administration of enzyme activity activators, could simulate the graft protective effects of IFLT in traditional cold preservation scenarios where ischemia-free liver transplantation technology is difficult to popularize.

#### 4.2.3 Clinical translation bottlenecks and emerging modalities

Despite the mechanistic consistency of the above combination strategies, their clinical translation still faces multiple bottlenecks. First, USP19 has two main isoforms: ER-anchored and cytoplasmic free-type, with different substrate spectra and functional division of labor: ER-anchored USP19 promotes migration and invasion in breast cancer (19), whereas the cytoplasmic isoform uc003cvz.3 exerts tumor-suppressive effects in ccRCC (8). Therefore, small-molecule modulators must address isoform selectivity and tissue targeting issues, avoiding “one-size-fits-all” interventions that lead to contradictory effects. Second, USP19 plays indispensable physiological functions in normal skeletal muscle, adipose tissue, and liver metabolic homeostasis: its gene knockout mice can resist muscle atrophy (29, 30), but systemic inhibition may disrupt normal tissue protein homeostasis and metabolic balance, causing off-target tissue toxicity (9, 31). Furthermore, currently no highly selective USP19 small-molecule modulators have entered clinical trials; existing combination strategies remain at the gene knockout/overexpression or cell/animal model level, with a significant gap remaining before human trials.

Emerging Modalities: PROTACs and Molecular Glues. Beyond conventional active-site inhibition, proteolysis-targeting chimeras (PROTACs) and molecular glues represent conceptually distinct approaches to modulate USP19 abundance. A PROTAC directed against USP19 would recruit an E3 ubiquitin ligase (such as VHL or CRBN) to induce USP19 proteasomal degradation. However, because USP19 itself possesses deubiquitinase activity, it may antagonize the ubiquitin signal required for PROTAC-mediated degradation; this intrinsic biochemical property suggests that PROTAC design would require either an irreversible ligand or allosteric targeting outside the catalytic domain. Isoform-selective PROTACs could exploit the ER-anchored transmembrane domain to restrict degradation to the membrane-resident variant, sparing cytosolic isoforms. Alternatively, molecular glues that stabilize the interaction between SIAH1/2 and USP19 could enhance USP19 ubiquitination and degradation, indirectly achieving functional inhibition. All these modalities remain purely conceptual; no USP19-targeting PROTAC or molecular glue has been reported, and their development depends on the prior discovery of high-affinity, isoform-discriminating ligands (**Table 2**).

**Table 2:** USP19 as a therapeutic target: intervention strategies across human diseases.

Disease	Functional role	Intervention direction	Molecular mechanism (substrate/linkage/key site)	Isoform involvement	Rationale for combined therapy	Emerging modalities	Evidence tier	Ref.
Glioblastoma	Oncogenic	Inhibition	Deubiquitinates/stabilizes MGMT → blocks proteasomal degradation → enhances O^6^-MeG DNA repair → TMZ resistance	Undefined	USP19 inhibitor + TMZ: dual blockade of DNA repair	PROTAC; molecular glue	Preclinical (genetic/cellular; xenograft)	(109)
Colorectal cancer	Oncogenic	Inhibition	Removes K48-linked chains from PD-L1 → blocks proteasomal degradation → maintains surface PD-L1 → immune evasion	ER-anchored	USP19 inhibitor + anti-PD-L1: dual targeting of PD-1/PD-L1 axis	PROTAC (membrane-permeable); molecular glue	Preclinical (genetic/cellular; immunocompetent mouse; organoid)	(68)
Hepatocellular carcinoma	Oncogenic	Inhibition	Deubiquitinates/stabilizes c-Myc and YAP → sustains glycolysis (Warburg effect) and proliferation	Undefined	USP19 inhibitor + antimetabolite/anti-angiogenic: dual metabolic and proliferative blockade	Isoform-selective small molecules	Preclinical (cellular/animal models)	(11, 121)
Diffuse large B-cell lymphoma	Oncogenic	Inhibition	Deubiquitinates/stabilizes PARK7 → activates Akt survival signaling → proliferation and G1/S progression	Undefined	USP19 inhibitor ± chemotherapy: dual suppression of survival signaling and cytotoxicity	PROTAC; molecular glue	Preclinical (cellular/animal models)	(101)
Cervical cancer	Oncogenic	Inhibition	Promotes p53 polyubiquitination and proteasomal degradation → loss of cell cycle arrest and apoptosis induction	Undefined	USP19 inhibitor + p53 restoration strategy: dual rescue of p53 tumor suppressor function	Molecular glue (enhancing SIAH-mediated USP19 degradation)	Preclinical (cellular models)	(41)
Gastric cancer	Oncogenic	Inhibition	Upregulates MMP2/MMP9 expression and gelatinase activity → ECM degradation → invasion and metastasis	Undefined	USP19 inhibitor + chemotherapy: dual blockade of invasion and proliferation	PROTAC	Preclinical (cellular; xenograft)	(113)
Triple-negative breast cancer	Oncogenic	Inhibition	Deubiquitinates/stabilizes LRP6 (Wnt co-receptor) → Wnt/β-catenin activation → migration/invasion	ER-anchored required	USP19 inhibitor + Wnt pathway inhibitor: dual blockade of metastasis	ER-anchored isoform-selective PROTAC	Preclinical (cellular; retrospective cohort)	(19)
Clear cell renal cell carcinoma	Tumor-suppressive (protective loss)	Activation	Loss of uc003cvz.3 → loss of ERK pathway suppression → proliferation/migration	Cytosolic (uc003cvz.3)	N/A (mono-restoration strategy)	Gene therapy (AAV-uc003cvz.3); small-molecule expression activators	Retrospective database; cellular (overexpression rescue)	(8, 10)
Alzheimer’s disease	Disease-promoting	Inhibition	Stabilizes NOX4 → ROS/ferroptosis → mitochondrial damage; Aβ-induced neuronal injury	Undefined	USP19 inhibitor + antioxidant/ferroptosis inhibitor: dual neuroprotection	Brain-penetrant PROTAC; molecular glue	Preclinical (cellular model only)	(42)
Pathological cardiac hypertrophy	Protective	Activation	Deubiquitinates TAK1 → suppresses TAK1 phosphorylation → attenuates p38/JNK1/2 → anti-hypertrophic/anti-fibrotic	Undefined	USP19 activator or SIAH1/2 interference: protein stabilization approach	Allosteric activator (relieving CS domain autoinhibition); molecular glue	Experimental (genetic models)	(1, 15)
Acute lung injury	Protective	Activation	Deubiquitinates TAK1 → inhibits TAK1 phosphorylation → reduces JNK/p38-driven inflammation and endothelial apoptosis	Undefined	USP19 activator or SIAH1/2 interference: anti-inflammatory protein stabilization	Allosteric activator; molecular glue	Experimental (genetic/LPS models)	(35, 37)
Hepatic ischemia-reperfusion injury after liver transplantation	Protective	Activation	Deubiquitinates SLC7A11 at K500 (removes K63-linked chains) → blocks autophagy-lysosomal degradation → preserves GSH synthesis and GPX4 activity → suppresses ferroptosis	Undefined	Recombinant USP19 protein or nano-delivery vector: exogenous supplementation	AAV vector; recombinant protein perfusion	Experimental (genetic/AAV models)	(43)

TMZ, temozolomide; O^6^-MeG, O^6^-methylguanine; GSH, glutathione; GPX4, glutathione peroxidase 4; LPS, lipopolysaccharide; AAV, adeno-associated virus. All intervention strategies represent mechanism-based hypotheses derived from preclinical genetic or cellular models. No selective small-molecule USP19 modulator has entered clinical trials. Strategies for ccRCC and AD are conceptual inferences based on disease mechanisms and lack *in vivo* intervention validation. Emerging modalities (PROTACs, molecular glues, allosteric activators) remain conceptual and require medicinal chemistry development.

Future validation of combination regimen efficacy and safety in organoid models, humanized mice, and large-scale patient-derived xenograft (PDX) models is needed, along with establishment of adaptive dosing standards based on USP19 expression levels.

## 5 Summary and outlook

USP19 is a modular deubiquitinating enzyme whose substrate-specific activity regulates protein stability, immune signaling, and cellular stress responses. Through its catalytic core and distinctive structural features—including N-terminal CS domains, a C-terminal transmembrane domain in specific isoforms, and intramolecular autoinhibition—USP19 participates in endoplasmic reticulum-associated degradation, innate immune attenuation, cell cycle control, and metabolic adaptation. Its pathological relevance spans neoplastic and non-neoplastic conditions, with functional outputs that depend critically on tissue context, isoform distribution, upstream transcriptional control, and immune microenvironmental cues.

USP19 stabilizes oncoproteins (c-Myc, YAP, PARK7), promotes immune evasion (PD-L1), and accelerates tumor suppressor degradation (p53) in multiple malignancies. In contrast, it suppresses excessive inflammation (TAK1-p38/JNK), protects against ferroptosis (SLC7A11), and maintains protein homeostasis in sterile injury and neurodegeneration. These opposing phenotypes are not contradictory; they reflect the differential availability of substrates, the subcellular localization of isoforms, and the divergent signaling demands of tumor cells versus stressed parenchyma. The immuno-regulatory hub hypothesis—positioning USP19 as a context-dependent switch between immune suppression and immune evasion—provides an integrative framework, though it remains to be tested in prospective, multi-tissue studies.

Several critical limitations constrain the translational readiness of USP19-targeted strategies. First, the functional redundancy between USP19 and other deubiquitinases (USP7, USP10, USP15, USP22) in p53, PD-L1, and MYC pathway regulation implies that selective inhibition may trigger compensatory activation, limiting efficacy. Second, the ER-anchored and cytosolic isoforms exhibit distinct, sometimes opposing, functions; small-molecule modulators must achieve isoform and tissue selectivity to avoid paradoxical outcomes. Third, USP19 is indispensable for normal skeletal muscle proteostasis, adipose metabolic balance, and hepatic phenylalanine metabolism; systemic inhibition or overexpression carries substantial off-target toxicity risk. Fourth, no selective small-molecule USP19 inhibitor or activator has entered clinical development, and all proposed therapeutic combinations remain at the genetic model or conceptual stage.

Future research should focus on the following directions. Structural biology efforts utilizing cryo-electron microscopy and X-ray crystallography to resolve USP19-substrate co-complexes would provide templates for rational drug design. Tissue-specific conditional Usp19 knockout models, combined with single-cell transcriptomics and proteomics, are needed to dissect cell-type-specific functions within complex microenvironments. The synergy between USP19 deficiency and anti-PD-L1 therapy in colorectal cancer warrants preclinical expansion into other microsatellite-stable solid tumors. In transplantation medicine, exogenous USP19 delivery—via recombinant protein or nano-vectors—could bridge the gap between ischemia-free and conventional preservation protocols. Systematic screening of upstream kinases, E3 ligases, and molecular chaperones that govern USP19 expression and activity may reveal more readily druggable indirect targets. Finally, large-scale prospective cohorts must validate whether USP19 expression, alone or within multi-gene signatures, can serve as a clinically actionable biomarker for diagnosis, prognosis, or treatment selection.

In conclusion, USP19 is positioned at the intersection of ubiquitin signaling, immune regulation, and tissue homeostasis. Its context-dependent functional duality poses significant biological and therapeutic challenges. Progress toward precision targeting will require not only chemical innovation to overcome selectivity and toxicity barriers, but also deeper mechanistic understanding of how the same deubiquitinase can protect one tissue while promoting malignancy in another.

## Glossary

AAV: Adeno-associated virus
ALI: Acute lung injury
ANP: Atrial natriuretic peptide
Akt: Protein kinase B
BAG6: BCL2-associated athanogene 6
Bax: BCL2-associated X protein
Bcl2: B-cell lymphoma 2
BNP: Brain natriuretic peptide
CD8⁺: Cluster of differentiation 8 positive
ccRCC: Clear cell renal cell carcinoma
Co-IP: Co-immunoprecipitation
CRC: Colorectal cancer
CTGF: Connective tissue growth factor
DLBCL: Diffuse large B-cell lymphoma
DMFS: Distant metastasis-free survival
DFS: Disease-free survival
Drp1: Dynamin-related protein 1
ER: Endoplasmic reticulum
ERAD: Endoplasmic reticulum-associated degradation
ERK: Extracellular signal-regulated kinase
GBM: Glioblastoma
GEO: Gene Expression Omnibus
GEPIA: Gene Expression Profiling Interactive Analysis
GPX4: Glutathione peroxidase 4
GSH: Glutathione
GSSG: Glutathione disulfide
GzmB: Granzyme B
HCC: Hepatocellular carcinoma
HSP90: Heat shock protein 90
IFLT: Ischemia-free liver transplantation
IFN-γ: Interferon gamma
IL: Interleukin
IRF4: Interferon regulatory factor 4
IRI: Ischemia-reperfusion injury
JNK: c-Jun N-terminal kinase
KPC1: Kip1 ubiquitination-promoting complex 1
LPS: Lipopolysaccharide
LRP6: Low-density lipoprotein receptor-related protein 6
MAPK: Mitogen-activated protein kinase
MAVS: Mitochondrial antiviral signaling protein
MDA: Malondialdehyde
ME1: Malic enzyme 1
MGMT: O⁶-methylguanine-DNA methyltransferase
Mfn1/2: Mitofusin 1/2
MMP2/9: Matrix metalloproteinase 2/9
NF-κB: Nuclear factor kappa B
NLRP3: NOD-like receptor family pyrin domain-containing 3
NOX4: NADPH oxidase 4
OS: Overall survival
PARK7: Parkinsonism-associated deglycase 7 (DJ-1)
PCNA: Proliferating cell nuclear antigen
PD-1: Programmed cell death protein 1
PD-L1: Programmed death-ligand 1
PDX: Patient-derived xenograft
PE: Phenylephrine
PI3K: Phosphoinositide 3-kinase
RORγt: Retinoic acid receptor-related orphan receptor gamma t
ROS: Reactive oxygen species
RCC: Renal cell carcinoma
shRNA: Short hairpin RNA
SLC7A11: Solute carrier family 7 member 11
SOD: Superoxide dismutase
TAC: Transverse aortic constriction
TAK1: Transforming growth factor-β-activated kinase 1
TBK1: TANK-binding kinase 1
TCGA: The Cancer Genome Atlas
Th17: T helper 17 cell
TMZ: Temozolomide
TNF-α: Tumor necrosis factor alpha
TRAF3: TNF receptor-associated factor 3
TRIF: TIR-domain-containing adapter-inducing interferon-β
TNBC: Triple-negative breast cancer
UPR: Unfolded protein response
USP: Ubiquitin-specific protease
USP19: Ubiquitin-specific protease 19
YAP: Yes-associated protein.
